# Effectiveness of the 10-Valent Pneumococcal Conjugate Vaccine (PCV-10) in Children in Chile: A Nested Case-Control Study Using Nationwide Pneumonia Morbidity and Mortality Surveillance Data

**DOI:** 10.1371/journal.pone.0153141

**Published:** 2016-04-08

**Authors:** Janepsy Diaz, Solana Terrazas, Ana L. Bierrenbach, Cristiana M. Toscano, Gizelton P. Alencar, Andrés Alvarez, Maria T. Valenzuela, Jon Andrus, Roberto del Aguila, Juan C. Hormazábal, Pamela Araya, Paola Pidal, Cuauhtemoc R. Matus, Lucia H. de Oliveira

**Affiliations:** 1 National Institute of Health, Santiago, Chile; 2 Sanas - epidemiologia e pesquisa, São Paulo, Brazil; 3 Department of Community Health, Institute of Tropical Pathology and Public Health, Federal University of Goiás, Goiânia, Brazil; 4 School of Public Health, University of São Paulo, São Paulo, Brazil; 5 Department of Statistics and Health Information, Ministry of Health, Santiago, Chile; 6 Medical School, University de los Andes, Santiago, Chile; 7 Sabin Vaccine Institute, Washington, DC, United States of America; 8 Pan American Health Organization, PWR-Chile, Santiago, Chile; 9 Comprehensive Family Immunization Project, Pan American Health Organization, Washington, DC, United States of America; Faculdade de Medicina de Lisboa, PORTUGAL

## Abstract

**Background:**

The ten-valent pneumococcal conjugate vaccine (PCV10) was introduced into the Chilean National Immunization Program (NIP) in January 2011 with a 3+1 schedule (2, 4, 6 and 12 months) without catch-up vaccination. We evaluated the effectiveness of PCV10 on pneumonia morbidity and mortality among infants during the first two years after vaccine introduction.

**Methods:**

This is a population-based nested case-control study using four merged nationwide case-based electronic health data registries: live birth, vaccination, hospitalization and mortality. Children born in 2010 and 2011 were followed from two moths of age for a period of two years. Using four different case definitions of pneumonia hospitalization and/or mortality (all-cause and pneumonia related deaths), all cases and four randomly selected matched controls per case were selected. Controls were matched to cases on analysis time. Vaccination status was then assessed. Vaccine effectiveness (VE) was estimated using conditional logistic regression.

**Results:**

There were a total of 497,996 children in the 2010 and 2011 Chilean live-birth cohorts. PCV10 VE was 11.2% (95%CI 8.5–13.6) when all pneumonia hospitalizations and deaths were used to define cases. VE increased to 20.7 (95%CI 17.3–23.8) when ICD10 codes used to denote viral pneumonia were excluded from the case definition. VE estimates on pneumonia deaths and all-cause deaths were 71.5 (95%CI 9.0–91.8) and 34.8 (95% CI 23.7–44.4), respectively.

**Conclusion:**

PCV10 vaccination substantially reduced the number of hospitalizations due to pneumonia and deaths due to pneumonia and to all-causes over this study period. Our findings also reinforce the importance of having quality health information systems for measuring VE.

## Introduction

In January 2011, the 10-valent pneumococcal conjugate vaccine (PCV10) was introduced in the Chilean National Immunization Program (NIP) with a 3-doses schedule at 2, 4, 6 months of age with a booster dose at 12 months, and with no catch-up vaccination for older children. Based on evidence from experimental and observational studies with the different-valent pneumococcal conjugate vaccines, vaccinated infants were expected to have reduced rates on a range of laboratory-confirmed and clinically-suspected pneumococcal disease morbidity and mortality outcomes.[1–8]

There are many reasons for assessing vaccine effectiveness (VE) and program impact (PI) of pneumococcal conjugate vaccines in post-licensure studies, which include measuring the benefit of herd-protection, the effectiveness of alternative vaccination schedules and the effect of changes in serotype distribution and antimicrobial resistance patterns. Vaccine effectiveness may differ in different settings. Observational study designs are usually needed to assess rare and long-term outcomes or absolute effects that are small.[9]

Population based observational studies provide an excellent approach for the measurement of VE.[10, 11] Retrospective studies using routinely collected surveillance data can usually be done rapidly and inexpensively, with the additional advantage that the data may be more representative of the vaccine target population.

The purpose of this study was to assess, in a nationwide nested case-control study, the association between PCV10 vaccination and pneumonia hospitalization and mortality (all cause and pneumonia related deaths) among infants in Chile, making use of four National individual-level surveillance databases.

## Material and Methods

### Study Design

This is a nested case-control study. Chilean children born in 2010 and 2011 exposed and not exposed to PCV10 vaccination were followed using routinely collected data and pneumonia hospitalization and mortality (all-cause and pneumonia related deaths) outcomes were assessed. Children entered the cohort at 2 months of age and were censored from the cohort when they died due to any cause, had any of the hospitalization outcomes or at the end of the two-years follow-up period. Using four case definitions, all cases were selected and four controls were randomly selected per case.

PCV-10 vaccination coverage was expected to have reached high levels in the target population right after it was introduced in the routine schedule at the beginning of 2011. In consequence of this high coverage, unvaccinated children born from 2011 onwards were expected to be not only rare, but they may also have had different characteristics from the vaccinated ones. For this reason, we sought the unvaccinated children from the 2010 live-birth cohort to act as an external comparison group for the vaccinated children from the 2011 live-birth cohort. Unvaccinated children born in 2011 were also included in the analysis, but they represented a small minority of all unvaccinated children included in the study.

### Ethical issues

The Ethics Committee of the Servicio de Salud Metropolitano Norte—Ministerio de Salud de Chile (CEI-SSMN 24/06/2013) approved this study. No informed consent was obtained as routine surveillance data sources were used, pertaining to the cohort of all 2010 and 2011 live births in Chile. After the record linkage procedures, records were anonymized and de-identified prior to statistical analysis.

### Study Population

All children born in Chile in 2010 and 2011 and registered in the National live-birth database were eligible to participate in the study. The annual birth cohort in Chile is ~250,000 children and all are expected to have a record in the National database. Chilean population in 2010 was 17.15 million.

### Data Sources and Data Cleaning

The study made use of four National case-based routinely collected surveillance databases: 1) Live-birth (2010, 2011), 2) Hospital discharge (2010-September 2014), 3) Mortality (2010-September 2014) and 4) Vaccination (2011, 2012). These databases were deterministically linked by means of a unique identification number specific to each individual in the country (Rol Único Nacional, known as RUT).

In the National live-birth database, apart from identification variables, hospital and date of birth, the following variables were available: borough of birth, whether the borough was in a urban/rural setting, gestational age, birth weight, mother’s number of children alive and dead and mother’s and father’s educational level.

The National Immunization Registry (RNI) is a new electronic on-line case-based database implemented in 2010, when it started recording vaccination campaigns, and expanded in January 2011 to include data from the routine vaccination schedule. Both public and private vaccination clinics are required to fill in the electronic on-line records for all vaccines included in the NIP. As a new notification system, the RNI was prone to two types of inconsistencies: 1) duplicates, i.e. two or more records representing the same vaccination episode, and 2) vaccination episodes that occurred in the first months in which the system operated may have been left out, without registration. With regards to the latter inconsistency, technical officers from the NIP did not expect these numbers to be great and, even if the first vaccination dose was not recorded for a particular child, there should be no reason for other doses not to be recorded. In order to identify and exclude duplicated vaccination episodes, a three-step process was applied. First, the RUT was used to link records belonging to the same individual. Secondly, as there were some records in which the RUT numbers were missing or markedly incorrect, the linkage strategy was complemented by means of an in-house deterministic record linkage algorithm, similar to the one validated by Pacheco et al,[12] which made use of the variables name of the child, date of birth, hospital and address. Next, duplicated records with the same or very similar dates of vaccination/registration were discarded, allowing for a maximum of four doses per child, with an interval of at least one month in between the doses.

The National Hospitalization Information System covered, in 2010 to 2013, 100% of public and > 90% of private hospitals in Chile, as informed by the Ministry of Health. All records that had International Classification of Diseases 10th Revision (ICD-10) codes for respiratory diseases (J00-J99) listed on the principal diagnosis field were available for analysis. There were duplicate hospitalization records, which were likely to be generated when cases were transferred within (e.g. one notification from the emergency room and another from the intensive care unit) or in between health care units. Similar to what was done in the RNI database, in the hospitalization database a three-step process was applied initially linking records by means of the RUT. We then considered that consecutive records of the same patient with a 14-day interval between discharge and re-entry belonged to the same episode of disease.[6, 13] Next, records of the same episode of disease were counted as representing only one hospitalization with the date of entry of the first one, but all ICD-10 codes listed on the principal diagnoses fields of each of them were kept for analysis.

In the National Mortality Information System, all records were available for analysis. There were two cause-of-death variables: one that was available to all records recording the ICD-10 codes for the underlying cause, and another that mostly referred to associate external causes of death, which was only completed for a negligible number of records. This later variable was not used in the analysis. Even though data were extracted in September 2014, the Ministry of Health warned the study team that there might have been deaths that occurred over the last months of 2013 –and therefore affecting mostly the vaccinated children—that had not yet been recorded in the database.

### Definition of cases and controls

The study was set up as a matched case-control nested on the cohorts of children born in 2011 (comprised mostly of unvaccinated children) and born in 2012 (comprised mostly of vaccinated children). Children entered the cohorts at the age of 60 days, corresponding to the scheduled date of the first dose of the PCV10 in the routine vaccination scheme. Children exited the cohorts upon completion of the two-years follow-up period or before that if they had died or had any other of the outcomes included in the four case definitions that were used:

1. Pneumonia hospitalizations or deaths: records with ICD-10 codes J12 to J18 listed as the principal discharge diagnosis or the underlying cause of death. Only one pneumonia event was considered per child. If the child had multiple pneumonia hospitalizations, only the first one was considered.
2. Bacterial or unspecified pneumonia hospitalizations or deaths: records with ICD-10 codes J13 to J18 listed as the principal discharge diagnosis or the underlying cause of death. Only one pneumonia event was considered per child. If the child had multiple pneumonia hospitalizations, only the first one was considered.
3. Bacterial or unspecified pneumonia deaths (ICD-10 codes J13 to J18 as the underlying cause of death).
4. All deaths.

Case definition number 2 was used to account for more pneumococcal pneumonia cases if children had been coded as having pneumonia due to bacteria or due to unspecified organisms (J13-J18) as opposed to viral pneumonia (J12). Importantly, the ICD-10 J12 code that denotes viral pneumonia is commonly used in Chile. The justification for this is that in Chilean emergency rooms and hospital wards, it is a very common practice to perform nasopharyngeal swab collections for multiple viral PCR-assays for children with upper and lower acute respiratory infections. The choice of code J12 for the principle diagnosis is probably based on test results rather than on clinical features.

For each of the four case definitions, four controls were randomly selected per case, matched on analysis time. The “sttocc” command in STATA 13 software was used to implement this step. Since all children entered the cohorts at 60 days of age, in this study analysis time was equivalent to age. This means that controls were alive and, for case definitions 1 and 2, had not been hospitalized with pneumonia at the time their matched cases had the outcomes studied. Moreover, vaccination status was ascertained for cases and controls at the time the cases had the outcomes studied.

### Ascertainment of exposure status

In Chile, all NIP vaccines are administered free of charge in local primary care units. When PCV-10 was first introduced in January 2011, the vaccination schedule had three doses administered at 2, 4, 6 months of age with a booster dose at 12 months (3+1 schedule), and with no catch up vaccination for older children. In 2012, the schedule was changed to two doses at 2 and 4 months of age with a booster dose at 12 months (2+1 schedule). However, this change is unlikely to have affected the present study, as children born from 2012 onwards were the ones who should have been submitted to the new reduced schedule, and they are not included in our analysis.

Vaccine status for children born in 2010 and 2011 was derived from the RNI database. Children were considered as vaccinated if they had at least one dose of recorded PCV10 vaccination. Children were considered as unvaccinated if they had no PCV10 vaccination recorded in the RNI database. For children classified as cases, vaccination status was ascertained at the age when they had the studied outcome. For children classified as controls, vaccination status was ascertained at the same age at which their matched cases had the studied outcome.

Some children born in 2010 may have had their vaccination status misclassified, to the effect that they may have been vaccinated but had not been recorded as such. As mentioned above, even though it is possible that the first dose of vaccination was not recorded for a few children born in late 2010 who reached 2 months of age right at the beginning of 2011—when the RNI database was starting the process of recording of the routine PCV10 scheduled doses—it is likely that they did receive this first dose and that all their subsequent doses should have been recorded. In other words, the vaccination status of such children would be misclassified mostly for cases that had outcomes in between 2 and 4 months of age and for their matched controls in the same age group.

Children born in 2010 may have also had their vaccination status misclassified due to other reasons. The 7-valent pneumococcal conjugate vaccine (PCV7) had been registered for use in Chile since 2009, but its use was mostly restricted to the private sector that, as per information obtained at the Ministry of Health, was expected to contribute with up to 5% of all infant vaccine doses. Additionally, vaccination of children considered to be at increased risk of pneumonia took place in 2009 and 2010 with PVC7. This concerned children born before 32 weeks of gestational age, under 1.5Kg or with lung dysplasia, which represent less than 0.5% of the total live-birth cohorts. As RNI started recording PCV10 vaccination in 2011, the study had no means of discriminating such children. In any case, it is likely that many of the children that received their initial unrecorded doses in 2010, either in the private sector or in this special program for children at increased risk of pneumonia, continued to receive subsequent doses in 2011, in which case they were recorded in RNI. In summary, vaccination status of children born in 2010 is likely to have been ascertained with some misclassifications, which happened more frequently for cases that had outcomes in younger ages and for their matched controls.

### Statistical analysis

Conditional logistic regression was used to obtain odds ratios for pneumonia hospitalizations and deaths in the vaccinated as compared to the unvaccinated children, taking into account the matching structure. Relevant confounders available in the live-birth database were adjusted for: sex, borough of birth in an urban/rural setting, mother’s education and gestational age. As there was evidence of collinearity in between father and mother’s education and in between birth weight and gestational age, father’s education and birth weight were excluded from the analysis.

Covariates were backwardly removed from the models one by one, beginning with the most weakly associated, based on their confounding effect and their contribution to the model, as assessed by the likelihood ratio (LR) test. Variables whose removal from the model caused substantial changes in IRR (>10%) were retained, as were variables whose removal incurred in significant LR tests (P <0.05).

All children registered in the live-birth database are presented in the descriptive analysis. However, in the regression analysis, those with a death record before completing 60 days of age and who had been wrongly recorded as having been vaccinated (158 children) or hospitalized before birth (42 children) were excluded. Vaccine effectiveness was calculated as (1- OR) x 100. The 95% confidence intervals were calculated similarly.

The Ministry of Health had warned the study team that, in the available data, there was a possibility of subnotification of deaths for the second semester of 2013, even though data extraction had taken place as late as September 2014. For this reason, when analyzing the case definitions 3 and 4, we ran separate models with a shorter follow-up of 18 months, instead of 24 months.

All analyses were done using STATA 13.

## Results

### Description of live-birth and vaccination registry data

**Fig 1:**
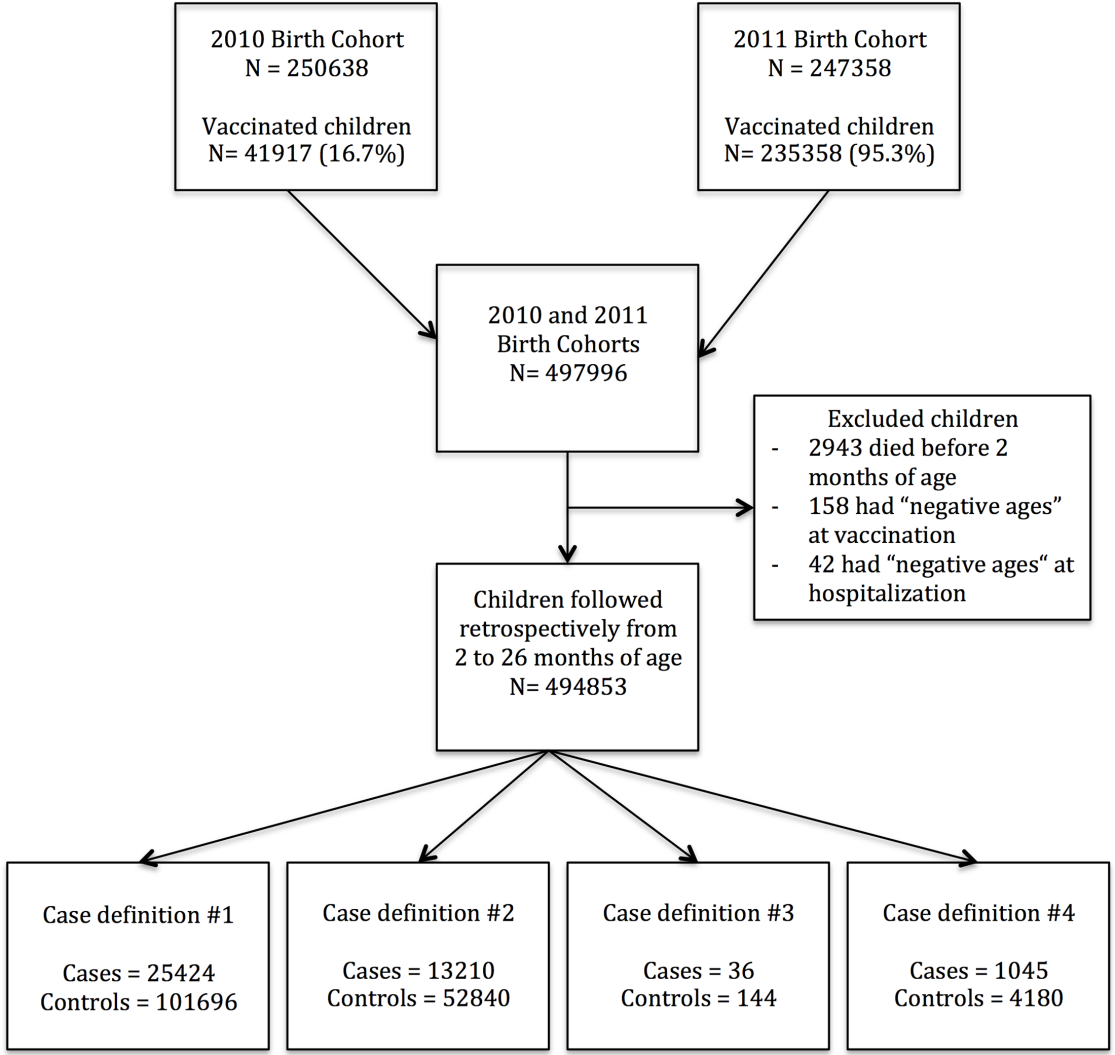
Flowchart of the selection of the study population.

There were 497,996 children born in Chile and registered at the National live-birth database: 250,638 born in 2010 and 247,358 born in 2011. A flowchart of the selection of the study population is shown in Fig 1.

In total, 787,344 pneumococcal vaccine doses were administered to a total of 281,892 children born in 2010 or 2011, with an average of 2.8 doses per children who received at least one dose. It is important to point out that by the time of data extraction, all children had aged sufficiently to receive all four doses of the vaccine, as recommended by the immunization programme.

Table 1 shows that the vast majority of vaccine doses administered were PCV-10. The other types were exclusively administered in private health centres and clinics.

**Table 1:** Types of pneumococcal vaccines administered in Chile to children born in 2010 or 2011.

Type of vaccine administered	N (%)
7-valent	94 (0.01%)
10-valent	769,761 (97.77%)
13-valent	16,542 (2.1%)
23-valent	947 (0.12%)
Total	787,344

Irrespective of the total number of doses received, the median age at vaccination was 71 days (IQR 63–132 days) for the first dose, 134 days (IQR 125–184 days) for the second dose, 270 days (IQR 190–377 days) for the third dose and 375 days (IQR 368–394 days) for the fourth dose. Even though most children received their doses close to the recommended ages (60 days, 120 days, 180 days and 365 days), there were several outliers representing unexpected ages at vaccination. At least for some of them, and of course for the 158 ones with “negative ages” at vaccination, wrong vaccination dates or even birth dates had been marked in their records, but they might actually have been vaccinated at the correct ages.

### Linkage between the live-birth and immunization registries

Of the total 250,638 children born in 2010, 41,917 (16.7%) were linked with the RNI, meaning that they received at least one dose of pneumococcal vaccine. These were mostly children born from September/2010 onwards who had two months of age from January/2011 onwards, when vaccination started targeting this age group. Of the total 247,358 children born in 2011, 235,358 (95.3%) were linked with the RNI, meaning that they had received at least one dose of pneumococcal vaccine.

There were a total of 4,147 records of children, born in 2010 or 2011 (as per information contained in the RNI), which were not linked to the live-birth database. At least some of these un-linked records must represent just extra doses with differently recorded RUTs (which were not recognized as belonging to the same person by means of nominal data—name, place and date of birth), of children whose other RNI records were properly linked with the live-birth database. Records of these children did not participate in the rest of the analysis.

### Description of hospitalization data

From January 2010 to February 2014, there were a total of 55,939 children, born in 2010/2011, registered in the hospitalization database as having been hospitalized at least once up to 26 months of age, due to any respiratory diseases (ICD-10 J codes). There were a total of 69,086 hospitalizations for these children. Of the total number of children, 80% had only one hospitalization and 1 of them had a total of 16. The mean number of hospitalizations per child who had been hospitalized at least once was 1.3 (SD 0.7). Of the 55,939 children, 57% were male and 43% were female.

Table 2 shows that almost half of all respiratory disease hospitalizations were due to pneumonia (J12-J18), and that the numbers due to viral pneumonia (J12) were a bit higher than those due to bacterial/unspecified pneumonia (J13-J18). Of the latter, only 56 were recorded with code J13, which refers to pneumococcal pneumonia, and over 83% were recorded with code J18, which refers to unspecified pneumonia.

**Table 2:** ICD-10 codes used for the principal hospitalization diagnosis for children aged 0 months to 26 months of age.

ICD- 10 codes used for principal diagnosis	N	%
J12 –Viral pneumonia	17,159	24.8
J13-J18 –Pneumonia due to bacteria, other infectious organisms and unspecified organisms*	16,356	23.7
J20 –Acute bronchitis	11,589	16.8
J21 –Acute bronchiolitis	8,842	12.8
Other respiratory codes	8,485	12.3
J00-J06 –Acute upper respiratory infections	5,886	8.5
J09-J11 –Influenza due to identified and unidentified influenza virus	769	1.1
Total	69,086	24.8

* J13—Pneumonia due to Streptococcus pneumoniae, J14—Pneumonia due to Haemophilus influenzae, J15—Pneumonia due to other specified bacteria, J16—Pneumonia due to other infectious organisms, not elsewhere classified, J17—Pneumonia in diseases classified elsewhere, J18—Pneumonia, unspecified organism.

**Fig 2:**
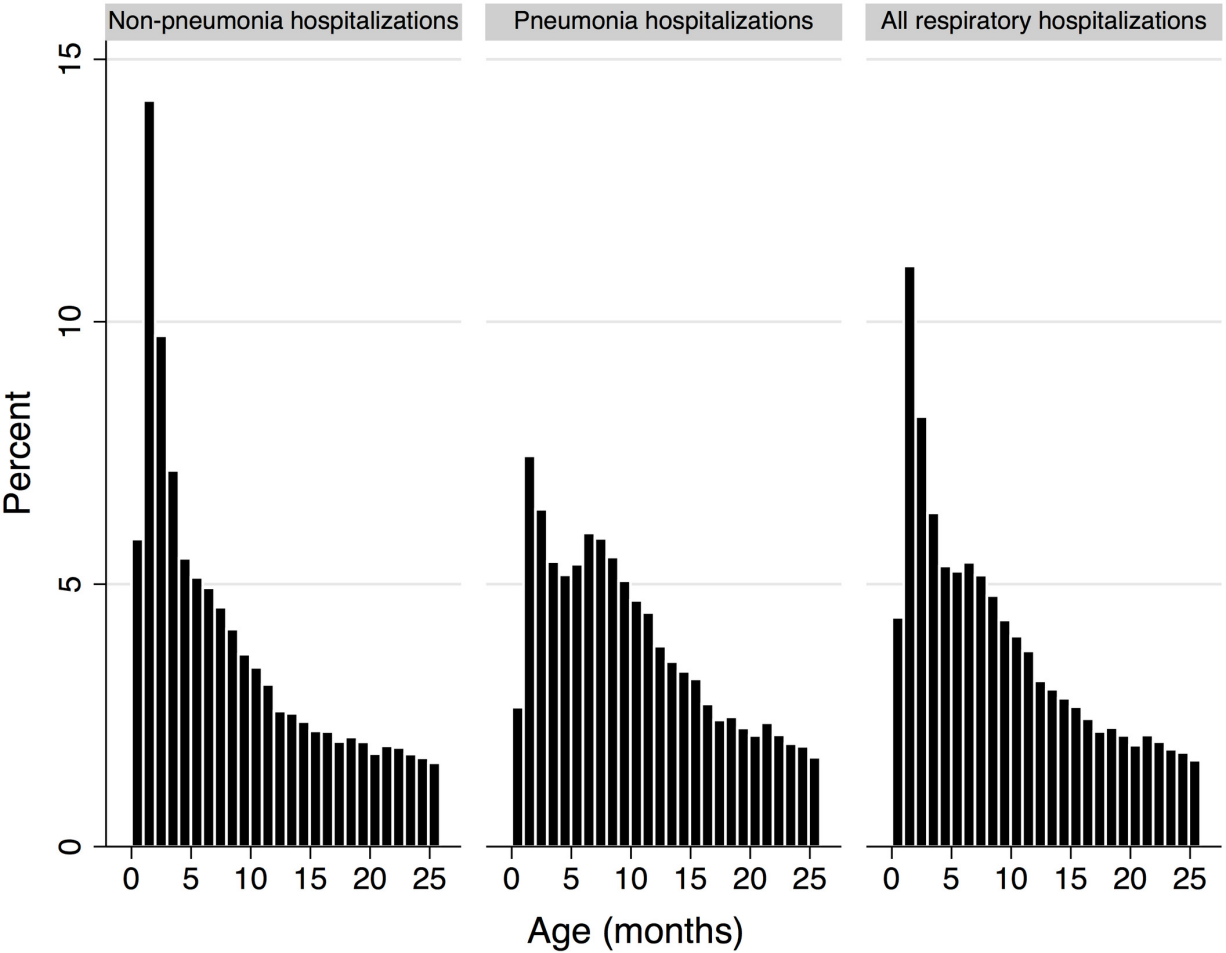
Age at hospitalization in Chile (months) for children up to 26 months of age born from January to September 2010.

Fig 2 shows that most non-pneumonia respiratory hospitalizations occurred in the first six months of life. Pneumonia hospitalizations were more spread out, but still most of them occur in the first year of life. As with other secondary databases, in this database there were also recording errors, as evidenced by 42 children who had “negative ages” at the time of their hospitalization. These children were excluded from the remainder of the analysis (and from Fig 1).

### Description of mortality data

Of the 497,996 in the live-birth database, 3991 (0.8%) died before completing 2 years of age. Of these 3991 deaths, 2943 (73.7%) died before completing 2 months of age. A total of 1048 children died from 2 months to 26 months of age, i.e. during the follow-up of the study. Among these deaths, only 50 were due to ICD-10 codes J12 to J18, 14 (28%) of which were coded as J12 and none was coded as J13. All 36 pneumonia deaths due to bacteria or to unspecified organisms (J13-J18) took place before 18 months of age. For children who died from 2 months to 26 months of age, causes of death listed under ICD-10 chapter XVII (Congenital malformations, deformations and chromosomal abnormalities) were the most frequent (408, 39.3%), followed by deaths listed under chapter XVIII (Ill-defined causes) (139, 13.4%). It is possible that at least some of these ill-defined deaths were actually due to pneumonia, but this could not be confirmed.

**Fig 3:**
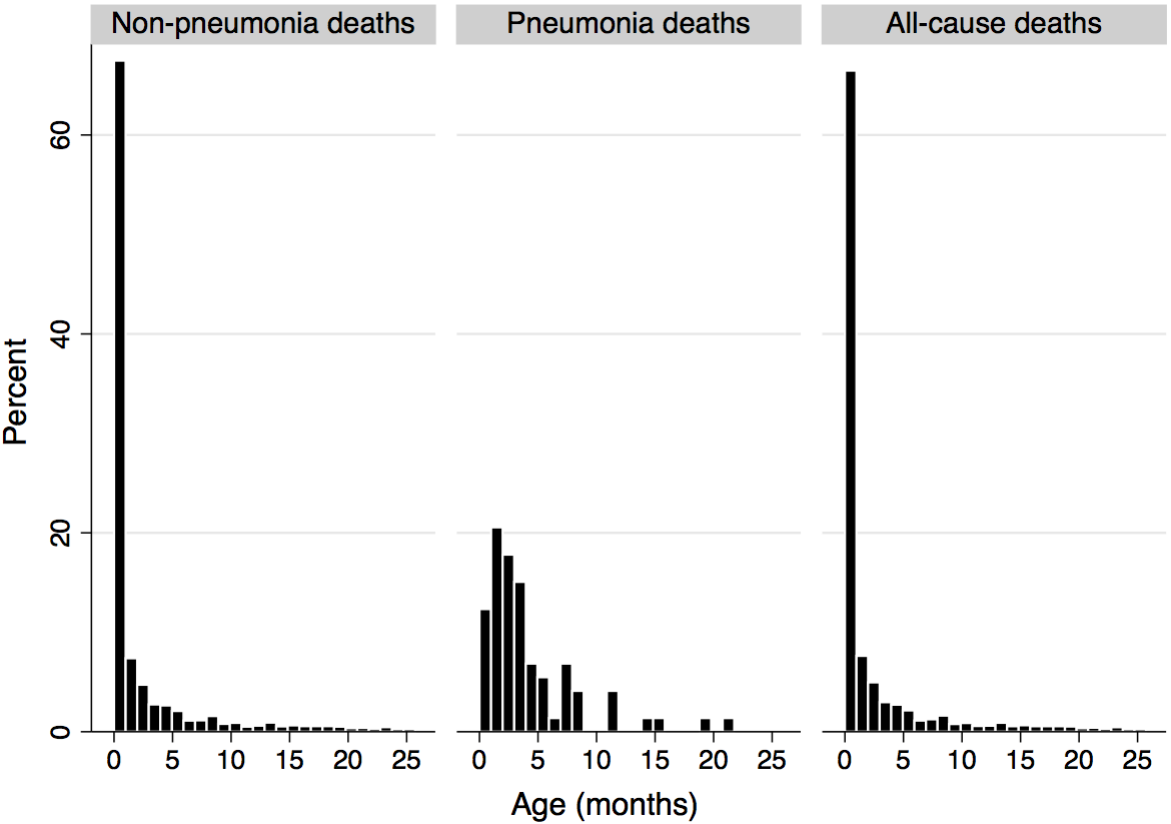
Age at death in Chile (months) for children up to 26 months of age born from January to September 2010.

Fig 3 shows that most deaths due to non-pneumonia causes occurred very early in the first month of life. Pneumonia deaths are more spread out, but the bulk of both pneumonia and non-pneumonia deaths occurred in the first six months of life.

### Comparison of cases and controls

Table 3 shows the numbers of vaccinated and unvaccinated children among cases and controls for the different case definitions, as well as the estimated adjusted odds ratios derived from the multiple regression analysis. Vaccine effectiveness was 11.2% (p-value<0.001) for the outcome of pneumonia hospitalizations and deaths (ICD-10 codes J12-J18). As expected, vaccine effectiveness increased when analysis only included ICD-10 codes for bacterial or unspecified pneumonia for both hospitalizations and deaths (20.7%, p-value <0.001). Estimated vaccine effectiveness for bacterial or unspecified pneumonia deaths was very high (71.5%, p-value = 0.048), and for deaths due to all it was 34.8% (p-values <0.001).

**Table 3:** Distribution of vaccinated and unvaccinated cases and controls and estimated adjusted vaccine effectiveness, for different case definitions.

Case definitions	Number of cases		Number of controls		Estimated effectiveness % (95%CI)*
	Vaccinated	Unvaccinated	Vaccinated	Unvaccinated	
1. Pneumonia hospitalizations and deaths—ICD-10 codes J12-J18	11,302	14,122	48,352	53,344	11.2 (8.5–13.6)
2. Bacterial/unspecified pneumonia hospitalizations and deaths—ICD-10 codes J13-J18	5,203	8,007	23,659	29,181	20.7 (17.3–23.8)
3. Bacterial pneumonia deaths—ICD-10 codes J13-J18	5	31	43	101	71.5 (9.0–91.8)
4. All deaths	344	701	1820	2360	34.8 (23.7–44.3)

* Models 1 and 2 were adjusted for sex, borough of birth in an urban/rural setting, mother’s education and gestational age. Models 3 and 4 were adjusted for mother’s education and gestational age.

Vaccine effectiveness estimates for bacterial pneumonia deaths and all deaths obtained in models with a shorter follow-up of 18 months were virtually identical to those obtained in models with a 24 months follow-up (data not shown).

## Discussion

In this nationwide nested case-control study of Chilean infants, PCV10 was estimated to have a protective effect on all-cause pneumonia hospitalizations and deaths, and this effect was somewhat increased when more specific case definitions were used. PCV10 was also estimated to have a substantial effect on pneumonia deaths as well as on deaths due to all causes.

For pneumonia hospitalizations, VE estimates are comparable—albeit on the lower end—to those presented in other observational studies in which the 3+1 schedule was used for different-valent vaccines, case definitions and study designs.[14, 15] A number of studies suggest that increasing the specificity of case definitions by applying more stringent and standardized diagnostic algorithms, including the use of radiological findings for example, increases estimates of vaccine effectiveness by increasing the number of pneumonias that are actually due to pneumococci.[4, 16, 17] Studies using routine surveillance data such as ours are contingent on the accuracy of ICD coding, i.e. on the extent to which the ICD codes reflect the underlying patients’ disease or cause of death. Diagnostic specificity is particularly relevant in places like Chile, in which the use of code J12 that denotes viral pneumonia is motivated, at least to a certain extent, by positive virology test results. However, significant morbidity due to pneumococci is associated with co-infection with viral respiratory infections, so that it is important to consider that such co-infection cases might be concealed under code J12, particularly when using surveillance data for the measurement of the burden of pneumococcal pneumonia.[18]

In Chile, 70% of pneumococcal pneumonias that occurred in the pre-vaccination period (2007 to 2010) in the study age group were due to serotypes included in the PCV10 vaccine.[19, 20] We do not know what proportion of pneumonia hospitalizations was due to pneumococcus, even though we assume that such proportion increased when we excluded code J12 (viral pneumonia) from our case definition. This could be the reason why VE increased when we restricted the range of pneumonia ICD-10 codes so as not to include code J12.

VE was also found to be higher against all cause mortality than against pneumonia hospitalizations and deaths. At least in part, this could be due to the proportion of pneumococcal pneumonias being higher among death records than among hospital discharge records, given that this aetiology is likely to be a more serious and life-threatening disease in infants than other viral and bacterial community acquired pneumonias requiring hospitalization. Moreover, among deaths coded with various underlying causes (such as death due to congenital malformations, deformations and chromosomal abnormalities or ill-defined causes), pneumonias, sepsis and/or meningitis due to pneumococci may have led or contributed to the fatal outcome.

But there may be another explanation as to why VE was high against all cause and also against pneumonia mortality. The mortality database provided by the Ministry of Health may not have included some deaths occurring in the last few months of 2013, so that some deaths may have been particularly missed in the vaccinated children. We knew from the pre-vaccination period data that most deaths in this age group occurred in the first six months of life, which were adequately represented in the database for both unvaccinated and vaccinated children. Therefore, we did not expect this bias to have a big influence on our results. In order to further appraise this influence, we ran models with shorter follow-up periods and obtained very similar VE results. Still, we acknowledge the fact that the addition of just a few unreported deaths to the vaccinated individuals would have reduced our VE estimates, in particular for pneumonia deaths.

We used a matched case-control analysis instead of a cohort survival analysis in order to avoid the immortal time bias.[21, 22] This type of bias, which favours the intervention group, would have happened if we had analysed our data as a cohort, because our outcomes could only have occurred in the vaccinated children if they had survived or had not had any pneumonia hospitalizations until their first vaccination. Vaccinated children would have a biased survival advantage—free from any outcomes—over the unvaccinated ones. The nested case-control design was able to avoid such problem, because cases were matched on analysis time to controls. Irrespective of vaccination status, it was guaranteed by design that cases and matched controls had been, by the time they were selected and their vaccination status was assessed, followed for an equal period of time.

However, other sources of bias might still have affected our VE estimates. Some children vaccinated in 2010 were misclassified as unvaccinated, at least for some time during their follow-up period. This misclassification probably affected less than 10% of the 2010 birth cohort and would tend to bias the measured estimate of VE towards the null.[11] Additionally, as an observational study that depended on routinely collected data, there might be residual confounding, despite the fact that our estimates were adjusted for the few covariates present in the live-birth database. We chose the cohort born in 2010 as our external comparison group from which most unvaccinated individuals were selected, because we believed that this cohort would be very similar in all respects to the cohort born in 2011, including in characteristics such as health care seeking behavior and access to health care. There was a strong earthquake in Chile in 2010, which has probably reduced the number of available hospital beds in the affected areas. However, Chile has a widespread network of public hospitals, so that the hospitals from the surrounding areas were capable of coping with the increased burden, and the overall number of pneumonia hospitalizations is not likely to have been affected by the earthquake. Because of this, we made the pre-hoc decision not to exclude the areas affected by the earthquake from our analysis.

This nationwide study was only possible in Chile because the country had invested in having strong surveillance and quality health information systems. The availability of case-based databases, which are easily merged by the presence of a common unique individual identifier number, should be encouraged in other countries. Moreover, case-based vaccination databases which to date have not been implemented in many low and middle-income countries, have multiple uses: from providing the means for the assessment of vaccine effectiveness, as in this study, to providing the means for the assessment of vaccine safety, supporting public health interventions to increase coverage and compliance, determining vaccination status to inform decisions by clinicians, schools and the health care system, and of course contributing to vaccine management and accountability. All of these are invaluable information in the context of the introduction of new vaccines to NIPs.[23]

In conclusion, PCV10 vaccination substantially reduced, in the target age group, the number of hospitalizations due to pneumonia and deaths due to pneumonia and to all-causes in Chile, as measured 3 years after vaccine introduction. Our findings also reinforce the importance of having quality health information systems for measuring VE.
